# Endoscopic Visualization of the Embolization Coil With Subsequent Endoscopic Therapy: A Case Report

**DOI:** 10.7759/cureus.58769

**Published:** 2024-04-22

**Authors:** Shahed Kamal, Karan Varshney, Sheng-Weng Lo, Vivekananda Ramachandran, Diana Lewis

**Affiliations:** 1 Department of Internal Medicine, Northern Hospital Epping, Melbourne, AUS; 2 Department of Public Health, School of Medicine, Deakin University, Waurn Ponds, AUS; 3 Department of Gastroenterology, Northern Health, Melbourne, AUS

**Keywords:** coil migration, over-the-scope clips, transarterial embolisation, gastro-intestinal, acute gastrointestinal bleed

## Abstract

Severe gastrointestinal bleeding is a common presentation to the emergency department. In such settings, trans-arterial embolization (TAE) may be conducted to address the bleeding. However, in some circumstances, this treatment may fail. Over-the-scope clips (OTSCs) have also shown efficacy when the first-line treatment is unsuccessful, and in this case report, we describe what we believe is the first reported application of OTSCs after TAE with partial coil migration. The patient had initially arrived at the emergency department with severe gastrointestinal bleeding, and despite the usage of inotropes and TAE, the patient had developed severe rebleeding. She ultimately recovered well after the utilization of OTSCs. This case report highlights that this form of management may be a valuable endoscopic therapy in preventing further coil migration for patients with emergency gastrointestinal bleeding.

## Introduction

There are numerous important forms of management for gastrointestinal bleeds in emergency settings. One valuable form of treatment that may be initiated in circumstances of gastrointestinal bleeding is the utilization of trans-arterial embolization (TAE) as a means to stop the bleeding. Studies have previously shown that the usage of TAE can be a safe and successful form of management for patients who may have lower gastrointestinal bleeds, as well as upper gastrointestinal bleeds; TAE may be done for patients who are unable to undergo surgery, but also in the management of postoperative hemorrhage [1-4]. However, in some cases, rebleeds may occur. In such circumstances, the usage of an over-the-scope clip (OTSC) may be recommended as a means of stopping the bleeds; this form of management has shown some effectiveness when the first-line forms of management fail [5]. However, to our knowledge, there have been no reports written on the usage of OTSCs post-TAE with partial coil migration.

In this report, we present the case of an elderly woman who presented to the emergency department with multiple episodes of hematemesis. After TAE was unsuccessful, OTSCs were used in a manner to effectively stop the patient's bleed, which has not been previously reported in the literature.

## Case presentation

A 76-year-old woman presented to the emergency department with hematemesis and melena at our hospital in Melbourne, Australia. She was brought in by ambulance after three acute episodes of hematemesis. Her past medical history included esophageal cancer, that had been previously treated with chemotherapy and radiotherapy. The patient also had a longstanding history of hypertension, type 2 diabetes, and chronic kidney disease. This patient had no known prior presentations of hematemesis. On presentation to the emergency department, her blood pressure was 142/105 mmHg, her heart rate was 105 beats per minute and her hemoglobin level was 90 g/dL (reference range in women: 12-15 g/dL). Upon presentation, there was a noted drop in her hemoglobin levels, and treatment of IV pantoprazole 80 mg BD was commenced, along with aggressive resuscitation with IV normal saline.

**Figure 1:**
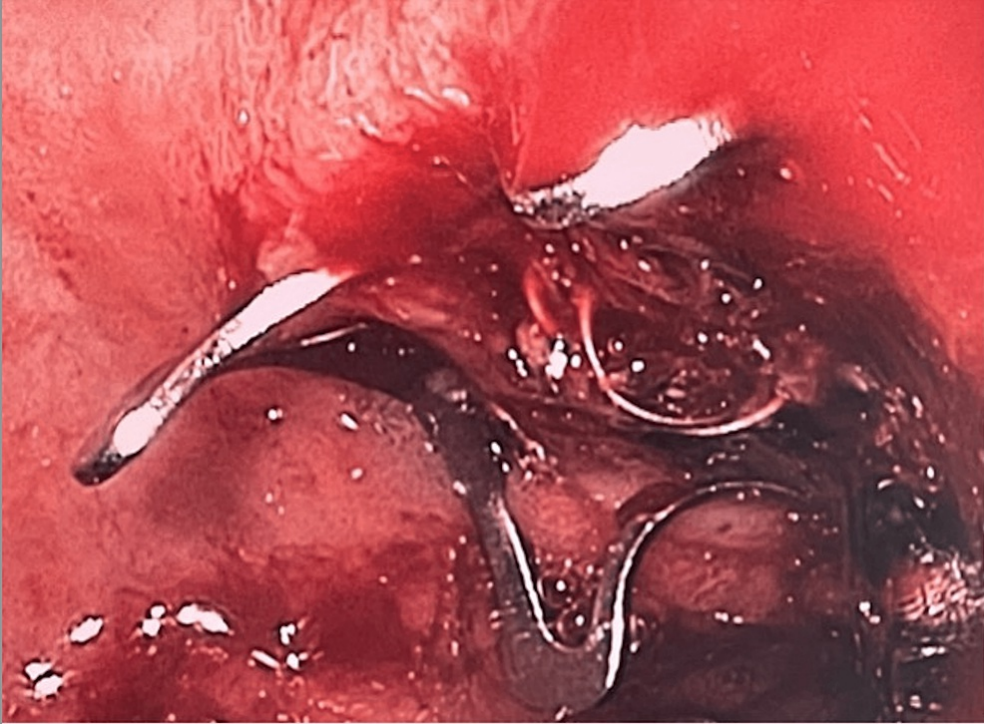
OTSC placed over coil and ulcer (though not completely closed) OTSC: over-the-scope clip

An emergent esophagogastroduodenoscopy (EGD) showed a Forrest Ia ulcer at the junction of the first and second part of the duodenum [6]. Endoscopic therapy with adrenaline injection into the Forrest Ia ulcer along with three clips (resolution) was performed. Although endoscopic hemostasis was achieved at the end of the session, there was concern about her very high risk of recurrent bleeding due to some of the ulcers being obscured by an unremovable clot. This prompted a TAE which found significant contrast extravasation from the gastro-duodenal artery in the region of the second part of the duodenum (Figure 1). 

**Figure 2:**
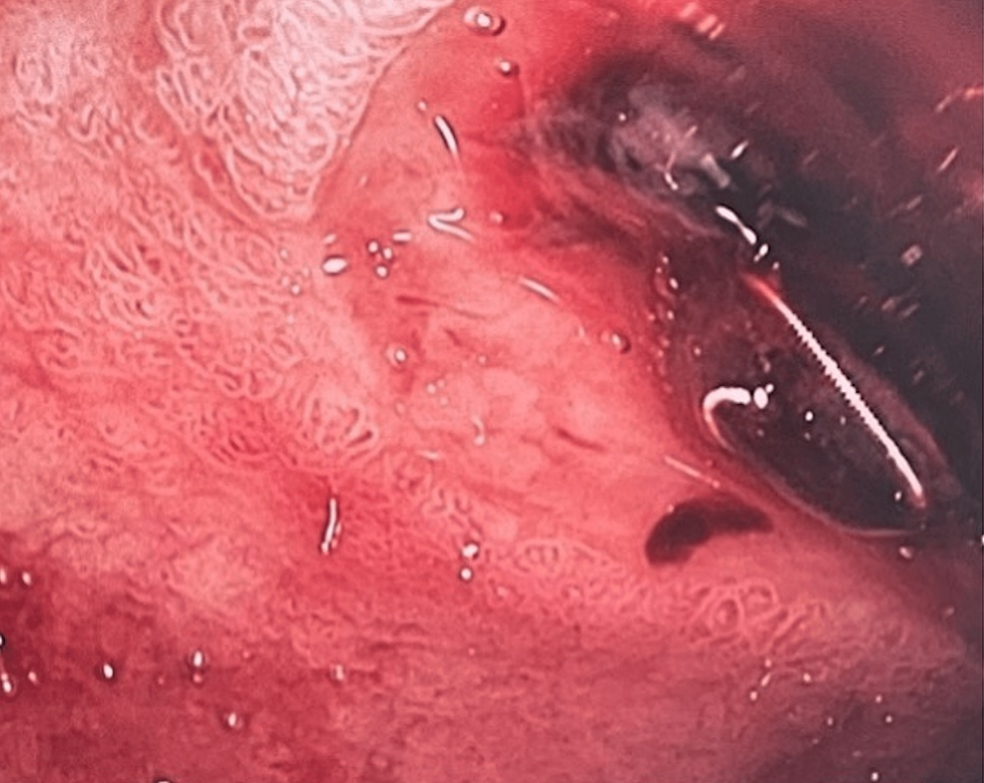
Active bleeding in the duodenum, with coil visible.

TAE with coils was subsequently performed with good angiographic results and showed no evidence of bleeding (Figure 2).

**Figure 3:**
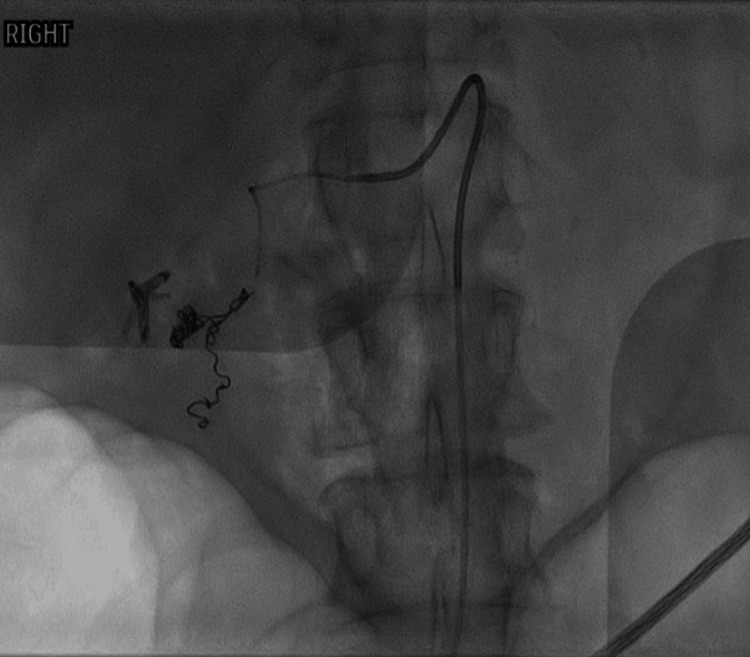
TAE with coils showing no evidence of bleeding. TAE: trans-arterial embolization

However, the patient had further episodes of hematemesis and melena and increased inotropic support whilst in the intensive care unit (ICU). The patient had become increasingly hemodynamically unstable at that point. A repeat EGD was performed in the ICU as the patient was too unstable for transfer for a repeat TAE. This showed active bleeding and a visible coil was seen in the duodenum (Figure 3).

**Figure 4:**
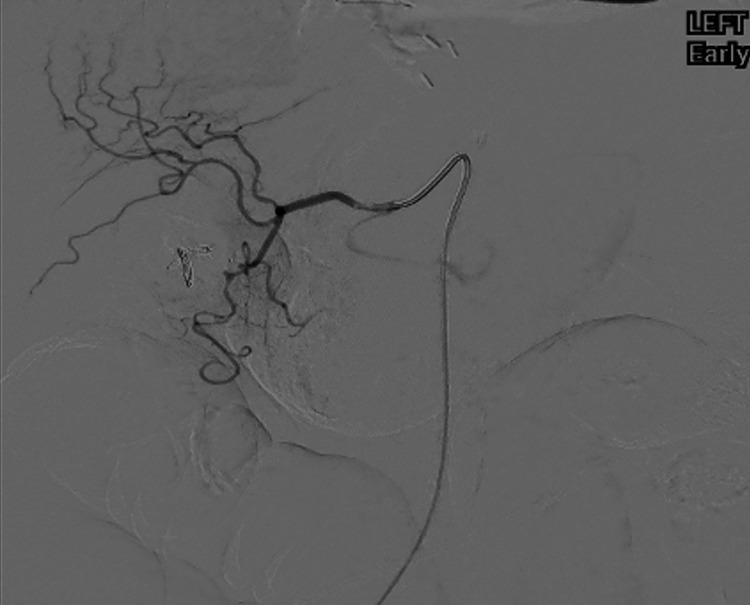
Image depicting significant contrast extravasation from the gastro-duodenal artery in the region of the second part of the duodenum..

An OTSC was placed over the coil and ulcer via endoscopy and hemostasis was achieved (although the OTSC did not fully close) (Figure 4).

The patient developed no notable complications thereafter. While the patient did initially show low iron and hemoglobin levels in the context of microcytic anemia, the patient was provided with packed red blood cells and iron transfusions over the next several days. Within a few days, her hemoglobin levels reached above 100 g/dL, and her iron had normalized. 

Despite the absence of major complications, the patient did have a prolonged hospital stay due to her functional decline given the multiple procedures on a background of frailty at baseline. While in the hospital, the patient underwent rehabilitation and had regular follow-ups, which involved collecting blood three times a week and strict bowel charting to monitor for any further bleeding. The patient did not develop any further bleeds during that time. One month after admission, the patient was discharged home and was scheduled to have a follow-up with a gastroenterologist for longer-term monitoring.

## Discussion

TAE is recommended for upper gastrointestinal bleeding when standard emergency endoscopic treatment fails due to rebleeding [7]. However, TAE has also been shown to have high rates of rebleed [8,9]. While OTSCs have shown efficacy when first-line treatment fails [5], to our knowledge, this is the first reported instance of applying OTSCs post-TAE with partial coil migration.

In this case, the patient was in a situation where they were experiencing severe, potentially life-threatening gastrointestinal bleeding. Despite initial attempts to address the bleeding, which included the usage of TAE, the patient's condition continued to worsen and she was transferred to the ICU. Ultimately, it was the usage of an OTSC that resulted in the stoppage of the bleeding, hence saving the patient's life. The findings of this report indicate that this could therefore prove to be a potential endoscopic therapy for preventing further coil migration in emergency settings; this is particularly relevant considering the high mortality rates associated with rebleeding following the failure of initial endoscopic treatment [10].

Past studies on the usage of OTSCs have shown encouraging results. For example, a prospective randomized control trial showed that endoscopic treatment that used OTSCs was superior to standard therapy in the management of recurrent peptic ulcer bleeding [11]. Similarly, a retrospective study demonstrated that OTSCs had similar success to surgical approaches for peptic ulcer bleeding that was refractory. However, OTSCs were also associated with lower in-hospital mortality, fewer adverse events, and a shorter stay in the ICU [12]. Similarly, of high pertinence is that a propensity score-matched analysis directly compared the effectiveness of OTSCs to TAE for refractory ulcer bleeding, and the study found that OTSCs were superior to TAE in terms of in-hospital mortality [7]. However, it is also worthwhile to denote that a different randomized control trial found that the usage of OTSCs in heavily bleeding peptic ulcers was not associated with a decrease in rebleeding after 30 days [13].

In consideration of the emergent studies highlighting the potential effectiveness of OTSCs, additional research is recommended to determine if OTSCs can address refractory gastrointestinal bleeds, and if it can encourage ulcer healing in high-risk patients. It will also be important for future studies to further evaluate the effectiveness of OTSCs compared to TAE in addressing rebleeding and to evaluate OTSC's effectiveness in cases where TAE has failed. In conclusion, our case highlights the use of the coil with the OTSC placed over it, which allowed hemostasis and eventual resolution of the patient’s condition. 

## Conclusions

In the case described here, a patient became unstable in the ICU after multiple attempts to address her gastrointestinal bleeding using standard forms of management, including inotropic agents and TAE. To our knowledge, this case report shows the first documented application of OTSCs post-TAE with partial coil migration. This may serve as a potential form of treatment in emergency gastrointestinal bleeds, and it is recommended that future studies assess the safety and effectiveness of this form of management in comparable clinical scenarios.
